# The Length of Lactation and Model of Weaning Modulate Key Regulatory Nodes of Murine Mammary Gland Involution

**DOI:** 10.3390/ijms262110501

**Published:** 2025-10-29

**Authors:** Sara Puebla, Amparo Gimeno, Elena Ortíz-Zapater, Rosa Zaragozá, Juan R. Viña, Elena R. García-Trevijano

**Affiliations:** 1 Departamento de Bioquímica y Biología Molecular, Facultad de Medicina, Universidad de Valencia, 46010 Valencia, Spain; sara.puebla@uv.es (S.P.); elena.ortiz-zapater@uv.es (E.O.-Z.); juan.r.vina@uv.es (J.R.V.); 2 Fundación Investigación Hospital Clínico-INCLIVA, 46010 Valencia, Spain; rosa.zaragoza@uv.es; 3 Departamento de Anatomía y Embriología Humana, Facultad de Medicina, Universidad de Valencia, 46010 Valencia, Spain; ampaparo.gimeno-monros@uv.es

**Keywords:** calpain-1, calpain-2, inflammation, lactation, mammary gland involution, miRnome, remodeling

## Abstract

Reduced lactation after forced weaning induces a tumor-promoting environment in the mammary gland, triggering key regulatory nodes common to both post-lactation involution and breast cancer, including calpains and miRs. We investigated how lactation duration modulates these nodes using two murine models: short lactation (forced weaning) and prolonged lactation (spontaneous-weaning). Additionally, the role of inflammation in calpain regulation was assessed in forced-weaned NOS-2 knockout mice. Morphological and molecular analysis in mammary tissue included histochemical staining, qPCR, enzymatic activity, Western blot and miRNA-seq. Mammary gland involution after prolonged lactation resulted in milder inflammation, reduced cell death and tissue remodeling, and lower collagen deposition compared to short lactation. The expression, activity and function of calpain-2 was found to be more sensitive to the model of lactation and inflammatory environment compared to calpain-1. Even after full regression (28 days postpartum), prolonged lactation maintained lower calpain-2 levels and higher expression of tumor suppressors miR-10b and miR-143/145. These persistent molecular differences suggest spontaneous weaning as the optimal model for healthy mammary gland regression, whereas forced weaning sustains alterations in calpain-2 and regulatory miRs that may increase post-partum breast cancer risk. The potential long-term influence of lactation duration on breast cancer development warrants further consideration in both clinical and public health contexts.

## 1. Introduction

It is widely accepted that the inflammatory environment triggered in the mammary tissue upon cessation of lactation can promote tumor progression [1,2,3]; the breastfeeding model seems to have an impact on the tumor promoting potential of the regressing mammary tissue after lactation [4,5]. Importantly, most women in developed countries shorten the period of lactation for work-related reasons. Accordingly, the study of breast cancer risk factors and underlying molecular mechanisms associated with the pregnancy/lactation cycle should consider the length of lactation.

In murine models, the interruption of lactation initiates the first phase of mammary gland involution; upon cessation of suckling stimulus, milk stasis and the drop in lactogenic hormones promote important changes leading to the inhibition of milk production and the induction of death in secretory cells [6,7,8]. After 72 h of weaning the mammary tissue enters into a p53-independent phase of involution. This is considered to be the second and irreversible phase, where the extracellular matrix (ECM) becomes disorganized, blood vessels are remodeled and adipocytes re-differentiate to regenerate mesenchymal tissue. Throughout this phase, a controlled influx of macrophages and other immune cells into the mammary gland clear dead milk-secreting cells, stimulating a pro-inflammatory environment [6,7]. Key regulatory nodes coordinate these sequential events to successfully return the mammary gland to a pre-pregnant-like state [8,9,10].

Both the duration of lactation and the model of weaning may have an impact on the onset of the second and irreversible phase of mammary gland involution [4,5]. The role of the regulatory nodes described for mammary gland involution has been studied only in experimental models of short lactation after the forced weaning of pups. Hence, breast cancer risk factors associated with the duration of lactation have been dismissed or under-observed.

Many proteins involved in post-lactation breast regression also play a key role in breast cancer progression when dysregulated [1,9,10]. Calpains are an example of regulatory nodes involved in the physiological involution of mammary gland after lactation [11,12,13], but also in breast cancer [1,14]. Calpain-1 and Calpain-2 belong to a family of Cys-proteases ubiquitously expressed in mammalian tissues [15]. Both calpains are heterodimeric proteases composed of an 80 KDa catalytic subunit (CAPN1 or CAPN2) and a common regulatory subunit (CAPNS1, also known as CAPN4) [3]. Since calpains are not degradative but processing enzymes, their function also extends to the functions of their multiple substrates or end-products [3,16]. As mentioned, the dysregulation of both calpains has been described in a plethora of diseases and cancer types including breast cancer [17,18]. However, the contribution of specific calpains to breast cancer risk is difficult to ascertain. The wide range of substrates, as well as the expression, activity or subcellular distribution of calpains in breast tissue can change under different conditions [11,12,13,16]. In addition, when the tissue microenvironment impacts a major regulatory node, it might trigger an amplified response across interconnected regulatory nodes such as miRs, ultimately influencing the downstream biological output in an exponential manner. Herein, we show that mammary gland regression is influenced by the model of lactation. Long lactation by spontaneous weaning seems to be the best choice for the mammary gland. In the short lactation model by forced weaning, persistent effects on regulatory nodes such as calpain-2, miR-10b and miR-143/145 may contribute to increased risk of postpartum breast cancer (PPBC). The long-term impact of these lactation-related effects on breast cancer risk should be considered.

## 2. Results

### 2.1. Morphological and Molecular Characterization of the Second Phase of Mammary Gland Involution in Forced and Spontaneous Weaning

**Figure 1:**
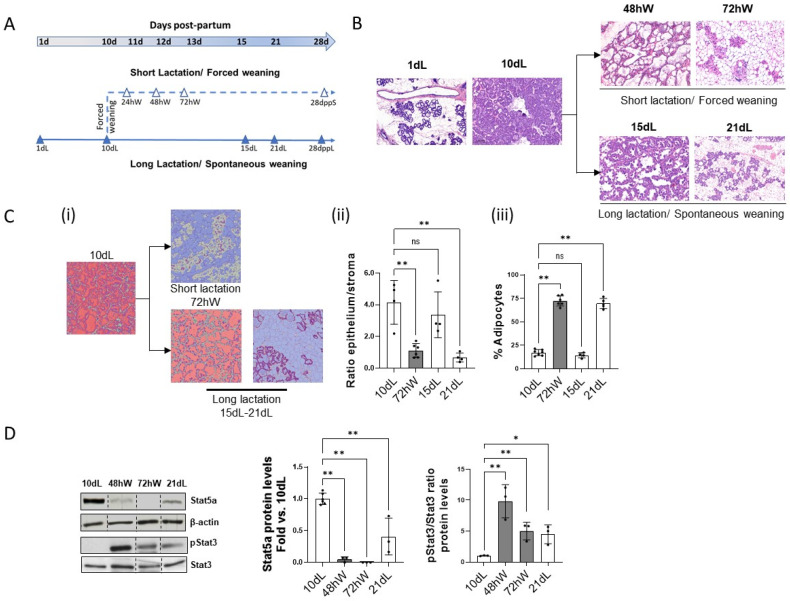
Characterization of the onset of second-phase mammary gland involution in different lactation models. (**A**) Experimental models of mammary gland involution. Time course for the collection of mammary tissue samples during lactation (filled triangles) and after forced weaning (open triangles) is shown. (**B**) Representative H&E-stained sections (n ≥ 3) at 1, 10, 15 and 21 days of lactation (1, 10, 15, 21 dL). In the short lactation model, mammary tissue was analyzed at 48 and 72 h after forced weaning (48 hW and 72 hW). (**C**) Representative H&E-stained tissue sections were scanned and computer-based labelled (**i**) to quantify the number of pink (epithelium), gray (stroma), or blue (adipocyte) staining pixels from the total number of pixels in the section. The epithelium-to-stroma ratio (**ii**) and percentage of adipocytes (**iii**) in 10 dL, 72 hW, 15 dL, and 21 dL was quantified and plotted. ** *p* < 0.01 vs. 10 dL. (**D**) Protein levels of Stat5a, pStat3 and Stat3 were analyzed by Western blot in 10 dL, 48 hW, 72 hW and 21 dL mammary gland extracts. Representative images are shown (n ≥ 3). Stat5a levels were quantified, normalized by β-actin and plotted as fold vs. 10 dL. Phospho-Stat3 and Stat3 levels were quantified and plotted as mean fold pStat3/Stat3 ratio vs. 10 dL. ** *p* < 0.01, and * *p* < 0.05. Non-adjacent lanes are indicated by lines. “ns” stands for not significant.

The effect of lactation length and weaning model on the onset of the second phase of involution was investigated. Two models of lactation were established as described in the Methods section and Figure 1A. In the well described short lactation model, pups were forced-weaned at the peak of lactation (10 dL) and mothers were sacrificed at the onset of the second phase of mammary gland involution (48 hW and 72 hW). For the long lactation model, pups lactated until spontaneous weaning, which occurs around 21 days after parturition. To examine how lactation duration influences the expression and function of regulatory nodes at the beginning of this phase, the onset of the second phase of involution in this model first needs to be defined.

The second phase of involution is characterized by massive epithelial cell death, basement membrane and ECM breakdown, and adipocyte repopulation of the mammary fat pad. The entrance into the second phase of involution in both models was assessed by H&E staining (Figure 1B). As already described [1], at 72 hW most epithelial cells are dead and the mammary fat pad is extensively repopulated by adipocytes. Apparently, the long lactation model exhibits a similar degree of regression at 21 dL. Indeed, the computer-based labeling of the tissue using QuPath (Figure 1C-i) commonly used to analyze the epithelial/stroma ratio (Figure 1C-ii) and adipocyte percentage (Figure 1C-iii), showed that the extent of alveolar regression and adipocyte repopulation at 21 dL is apparently the same as at 72 hW.

Finally, although the changes in the marker of lactation (STAT5) at 21 dL are milder than at 72 hW, the marker of involution (pSTAT3/STAT3) and the whole analysis reveal that involution was already initiated at 21 dL (Figure 1D).

### 2.2. Effect of the Duration of Lactation on Calpain Expression and Activity During Mammary Gland Involution

**Figure 2:**
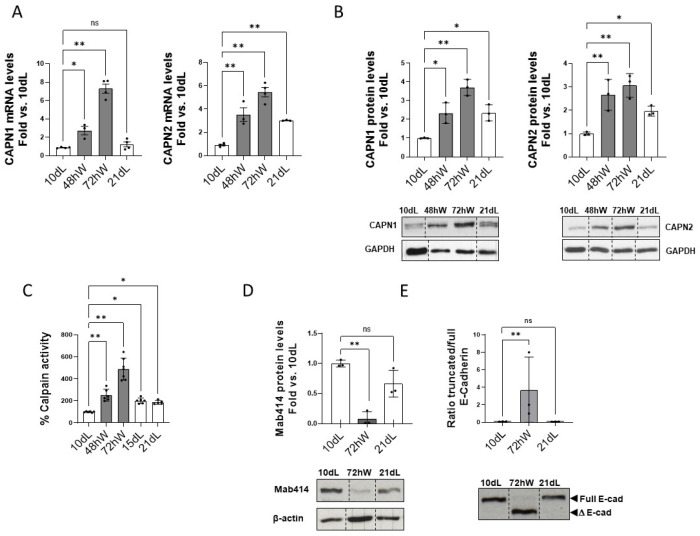
Effect of the model of lactation on calpains expression, activity and function during mammary gland involution. (**A**) mRNA levels of CAPN1 and CAPN2 were analyzed by qPCR in mammary gland tissue samples at 10 dL, 48 hW, 72 hW and 21 dL. Data were normalized by Ipo8 mRNA levels and plotted as mean (n ≥ 3) fold vs. 10 dL. (**B**) CAPN1 and CAPN2 protein levels analyzed by Western blot in mammary gland samples at 10 dL, 48 hW, 72 hW and 21 dL. Quantified data (n ≥ 3) were normalized by GAPDH and plotted as fold vs. 10 dL. (**C**) Calpain enzymatic activity in mammary gland samples (n ≥ 3) at 10 dL, 48 hW, 72 hW, 15dL and 21 dL plotted as % activity vs. 10 dL. Proteolysis of nucleoporins (**D**) or E-cadherin (**E**) was analyzed by Western blot with Mab414 and anti-E-Cadherin antibodies, respectively. Full length nucleoporin was quantified, normalized by β-actin and plotted as mean (n ≥ 3) fold vs. 10 dL. Full and truncated E-cadherin were quantified and plotted as the ratio truncated/full E-cadherin. vs. 10 dL. Representative images are shown (n ≥ 3), ** *p* < 0.01 and * *p* < 0.05. Non-adjacent lanes are indicated by lines. “ns” stands for not significant.

The expression of both calpains has been reported to peak at the beginning of the second phase of mammary gland involution after forced weaning [11,12,13]. Accordingly, 72 hW and 21 dL samples were selected to explore the effect of duration of lactation on CAPNs expression, activity, and function during the irreversible phase of mammary gland involution compared to the peak of lactation (10 dL). Interestingly, during mammary gland involution the increase of mRNA and protein levels of both CAPN1 and CAPN2 was milder in the long (21 dL) compared to the short (72 hW) lactation model (Figure 2A,B).

Moreover, expression levels of both calpains at 21 dL were the same as those at 48 hW or even lower. Since a direct correlation between expression levels and activity or function of calpains cannot be assumed [1,12,13] we ensured to detect any potential difference affecting their enzymatic activity or function on target proteins. In agreement with RNA and protein levels, the short lactation model exhibited much higher enzymatic activity at 72 hW (Figure 2C) than spontaneous weaning at 15 dL and 21 dL.

The role of the lactation model on the function of CAPN isoforms was studied analyzing the cleavage of those proteins known to be the isoform-specific targets of calpains during mammary gland involution [11,12,13]. The CAPN1-mediated cleavage of nucleoporins and the cleavage of E-cadherin by CAPN2 were compared in both lactation models. As shown in Figure 2D,E, although both CAPN-targets were cleaved at 72 hW, this cleavage was not observed at 21 dL.

On the whole, these results suggest that the duration of lactation affects the expression, activity and function of CAPNs.

### 2.3. Role of the Inflammatory Response on the Modulation of Calpains During Mammary Gland Involution

**Figure 3:**
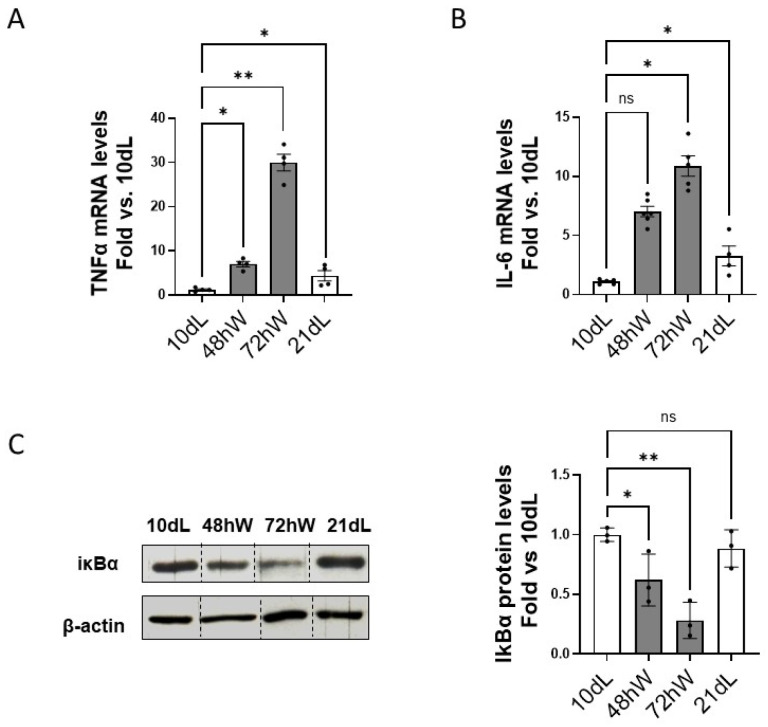
Effect of duration of lactation on the inflammatory environment at the onset of mammary gland involution. mRNA levels of (**A**) TNFα and (**B**) IL-6 were analyzed by qPCR in 10 dL, 48 hW, 72 hW and 21 dL mammary gland tissue samples. Data were normalized by Ipo8 mRNA levels and plotted as mean (n ≥ 3) fold vs. 10 dL ** *p* < 0.01 and * *p* < 0.05. (**C**) Proteolysis of IκBα was analyzed by Western blot in mammary gland extracts at the indicated time points. Quantified data (n ≥ 3) were normalized by β-actin and plotted as fold vs. 10 dL ** *p* < 0.01, * *p* < 0.05. Representative image is shown. Non-adjacent lanes are indicated by lines. “ns” stands for not significant.

It has been documented that abrupt weaning in FVB/N mice induces a sustained inflammatory response in the mammary tissue lasting for 7 weeks after weaning [4,5]. We wondered whether the differential expression of both calpains could be supported by a different inflammatory response at the onset of the second phase involution in both lactation models. The expression of TNFα and IL-6 were indeed dramatically higher at 72 hW than at 21 dL (Figure 3A,B). The immunomodulatory transcription factor NFκB is known to regulate the expression of both calpains during mammary gland involution after forced weaning [9,11]. Remarkably, the levels of IκBα which inversely correlate with the activation of NFκB, remained unchanged from 10 dL to 21 dL (Figure 3C) indicating that the activation of this factor was inhibited or delayed during spontaneously induced mammary gland involution.

To specifically study the contribution of the inflammatory environment to the modulation of calpains at the second phase involution NOS-2 KO mice were used. Although genetic modifications may influence other pathways beyond inflammation, we previously reported a milder inflammatory response in mammary tissue of forced-weaned NOS-2 KO mice [19]. While mammary gland regression was completed in these animals, its entrance into the second phase of involution was delayed. During post-lactation involution in KO mice the mild additive effect of NOS-1 and NOS-3 induction observed at 48 h–72 hW explains the reduction—but not the absence—of nitrites in these animals. Nitrite levels in 24 hW NOS-2 KO mammary gland did not reach the threshold observed in WT mice [19]. Consequently, entrance into the second phase of involution was delayed by 24 h (Figure S1).

**Figure 4:**
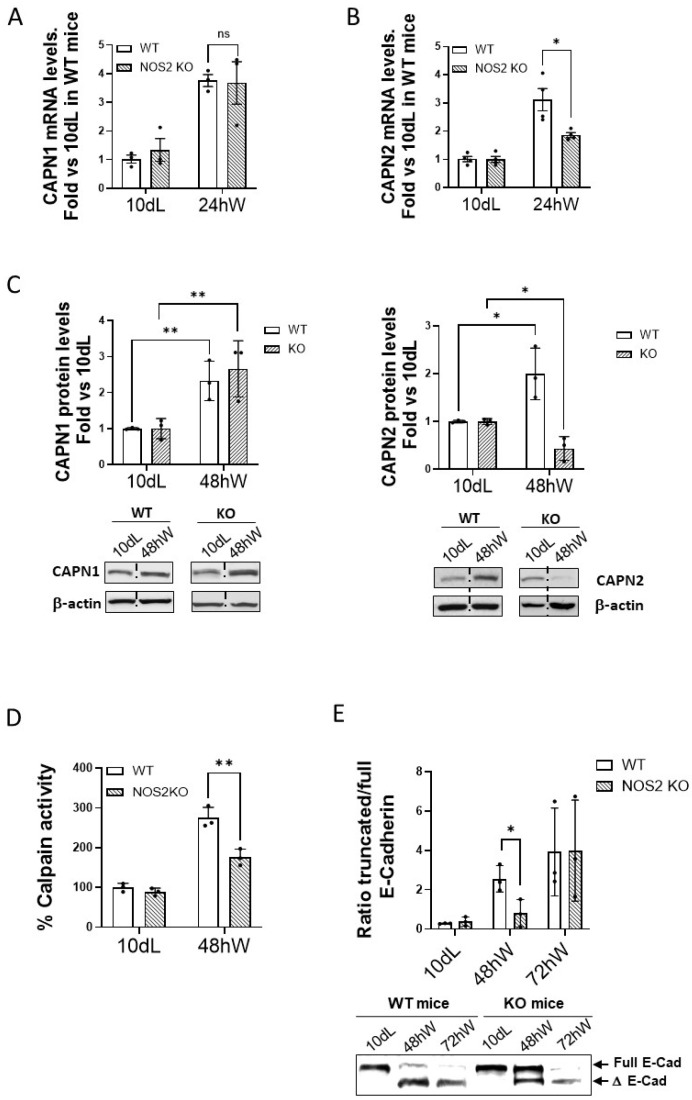
Effect of the inflammatory environment on calpains during mammary gland involution. mRNA levels of CAPN1 (**A**) and CAPN2 (**B**) analyzed by qPCR in mammary gland tissue samples from WT (white) and NOS-2 KO (dashed) mice at 24 hW. Data (n ≥ 3) were normalized by Ipo8 mRNA levels and plotted as mean fold vs. 10 dL. * *p* < 0.05. (**C**) CAPN1 (**left**) and CAPN2 (**right**) protein levels analyzed by Western blot in 10 dL and 48 hW mammary gland samples from WT (white) and NOS-2 KO (dashed) mice. Quantified data were normalized by β-actin and plotted as mean (n ≥ 3) fold vs. 10 dL * *p* < 0.05. and ** *p* < 0.01. Representative images are shown. (**D**) Calpain enzymatic activity in 10 dL and 48 hW mammary gland samples from WT (white) and NOS-2 KO mice (dashed). Data were plotted as percentage (n ≥ 3) vs. 10 dL in WT mice. ** *p* < 0.01 KO mice compared to WT at 48 hW. (**E**) Proteolysis of E-cadherin analyzed by Western blot in 10 dL, 48 hW and 72 hW mammary gland samples from WT (white) and NOS-2 KO mice (dashed). Full and truncated E-cadherin were quantified and plotted as the mean (n ≥ 3) ratio truncated/full E-cadherin. * *p* < 0.01 KO mice compared to WT at 48 hW. Representative images are shown. Non-adjacent lanes indicated by lines. “ns” stands for not significant.

Interestingly, the expression of CAPN2 seemed to be more sensitive than CAPN1 to the delayed response observed in the NOS-2 KO mammary gland. While this 24 h delay did not affect the mRNA levels of CAPN1 (Figure 4A), induction of CAPN2 mRNA levels was significantly delayed in NOS-2 KO compared to WT mice (Figure 4B).

CAPN2 protein levels and calpain enzymatic activity were also reduced in 48 h weaned mammary tissue from NOS-2 KO mice when compared to their WT mice counterpart (Figure 4C,D). In agreement with these findings, a modest cleavage of E-cadherin was observed at 48 hW in NOS-2 KO compared to WT mice (Figure 4E), indicating that calpain-2 function was also compromised (Figure S2).

These data suggest that the modulation of (at least) CAPN2 expression, activity and function is affected by NO levels and the inflammatory response in mammary tissue during forced weaning involution.

### 2.4. Effect of the Duration of Lactation on Mammary Tissue Remodeling

We already reported a decreased expression of procollagens (V)a1, (XIV)a1, (III)a1 and Lysil oxidase in mammary gland samples from NOS-2 KO mice after forced weaning [19]. In addition, an inflammation-mediated role for calpains in the organization of ECM in several in vivo and in vitro experimental models has previously been published [20,21]. The differences found in the inflammatory response of the mammary gland according to the lactation model could have an impact on breast remodeling during the second phase of involution. Thus, mammary tissue remodeling was analyzed as means of metalloproteinases (MMP9 and MMP2) and TIMP-1 expression, Masson’s trichrome staining, and percentage of adipocytes in mammary tissue sections.

**Figure 5:**
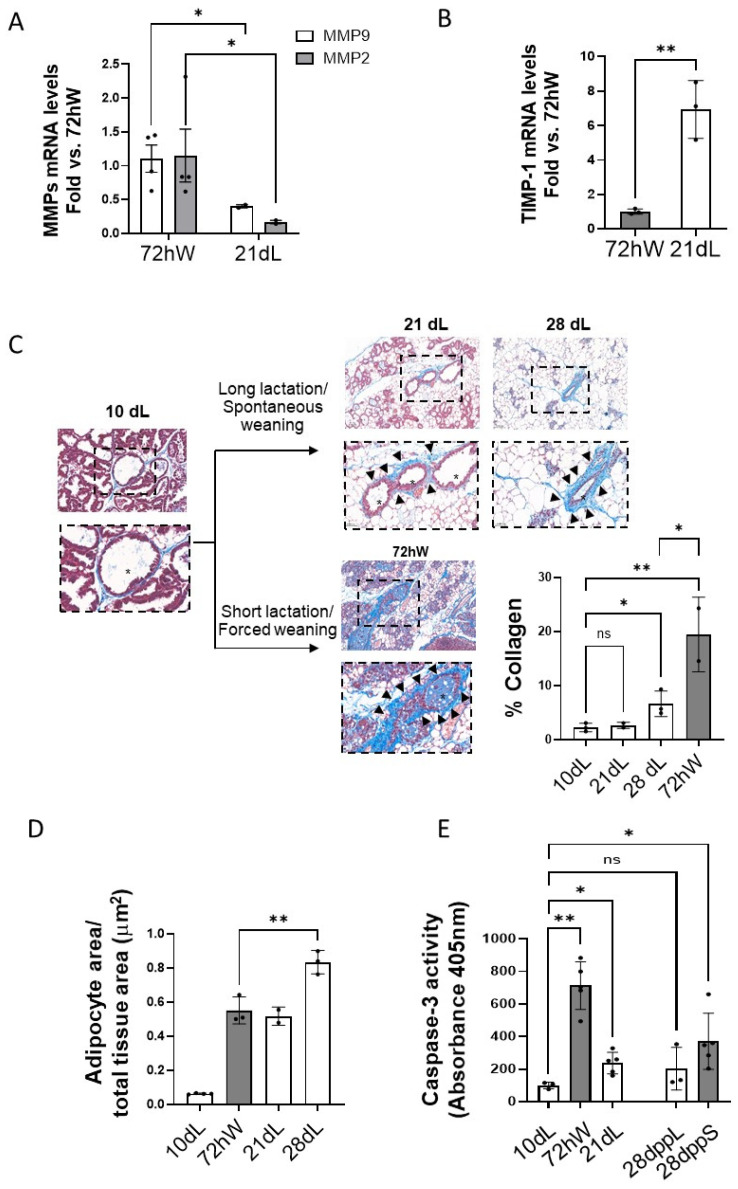
Effect of the duration of lactation on mammary tissue remodeling. mRNA levels of (**A**) MMP9 (white), MMP2 (gray) and (**B**) TIMP-1 in mammary tissue at 72 hW and 21 dL analyzed by qPCR were quantified, normalized by Ipo8 and plotted as mean fold vs. 72 hW (n ≥ 3) ** *p* < 0.01, * *p* < 0.05. (**C**) Trichrome-stained mammary tissue sections harvested at 10 dL, 21 dL, 28 dL and 72 hW, (n ≥ 3) were scanned, quantified and plotted as % collagen. ** *p* < 0.01 and * *p* < 0.05 vs. 10 dL. (**D**) Adipocyte area/total tissue section area in mammary gland samples (n ≥ 3) collected at 10 dL, 21 dL, 28 dL and 72 hW was quantified and plotted. ** *p* < 0.01. n = 4/tissue section. (**E**) Caspase-3 activity in mammary gland samples (n ≥ 3) at 10 dL, at the onset of second phase involution (72 hW and 21 dL) and at full regression of mammary gland at 28 days post-partum in the long (28 dppL) and the short (28 dppS) lactation models. * *p* < 0.05, ** *p* < 0.01 vs. 10 dL. “ns” stands for not significant.

Consistent with a milder inflammatory environment in the long lactation model, a higher MMP-to-TIMP-1 ratio (Figure 5A,B) and collagen deposition (Figure 5C) was observed in mammary gland at 72 hW compared to 21 dL.

It has been reported that the lower the MMP/TIMP-1 ratio the slower the involution [22]. Accordingly, remodeling could be delayed in the long lactation model. To explore whether this was the case, mammary gland samples in the long lactation model were also examined at 28 dL. As shown in Figure 5C, collagen deposition was also lower at 28 dL than at 72 hW. On the contrary, the percentage of adipocytes repopulating the mammary fat pad after spontaneous weaning (28 dL) was higher than at 72 hW (Figure 5D). These data might indicate that spontaneous weaning after long lactation does not just delay the start of the second phase of involution, but rather induces a milder and efficient response in the mammary tissue to return it back to the virgin-like state. Alternatively, the difference in days post-partum could account for the differences found in both models.

Caspase-3 activity known to peak at the first phase of mammary gland involution was analyzed in both models (Figure 5E). This analysis also included mammary gland tissue samples at the same days post-partum for both short (28 dppS) and long (28 dppL) lactation models following the experimental design represented in Figure 1A. As expected, caspase-3 activity showed a marked increase at 72 hW, followed by a decline at 28 dppS. In contrast, a milder yet sustained elevation in caspase-3 activity was observed at 21 dL, with no further increase at 28 dppL. These findings support the notion that the second phase of involution in this model was not merely delayed but followed a milder pattern.

All these data indicate that weaning after short lactation might induce an acute response with dramatic changes in the organization of mammary tissue that might end up into an altered tissue and ECM structure.

### 2.5. Effect of the Lactation Model on Regulatory Nodes upon Full Regression of Mammary Gland

The milder response of the mammary gland to spontaneous weaning might have important consequences for the complete regression of the mammary tissue. Alternatively, the mammary microenvironment might converge in both models at the end of post-lactational involution with no major consequences. We studied whether the microenvironment of the fully regressed mammary gland influences the expression, activity, and function of calpains.

**Figure 6:**
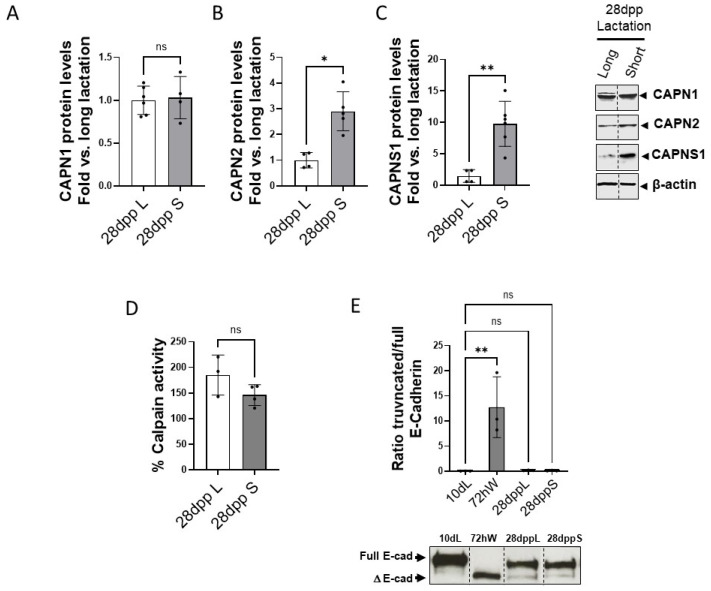
Calpains changes in the mammary tissue at 28 days postpartum in the long/short lactation models. Protein levels of (**A**) CAPN1, (**B**) CAPN2 and (**C**) CAPNS1 analyzed by Western blot in mammary gland samples at 28 dppL and 28 dppS. Quantified data (n ≥ 3) were normalized by β-actin and plotted as mean fold vs. 28 dppL ** *p* < 0.01 and * *p* < 0.05. Representative images are shown. (**D**) Calpain enzymatic activity in mammary gland samples (n ≥ 3) at 28 dppL and 28 dppS. No significant differences were found (n.s.). (**E**) Proteolysis of E-cadherin was analyzed by Western blot in 10 dL, 72 hW, 28 dppS and 28 dppL. Full and truncated E-cadherin (ΔE-cad) were quantified and plotted as the mean (n ≥ 3) ratio truncated/full E-cadherin. ** *p* < 0.01 vs. 10 dL. Representative images are shown. Non-adjacent lanes indicated by lines. “ns” stands for not significant.

Interestingly, although the mRNA levels of CAPN1, CAPN2 and CAPNS1 at 28 dpp were not statistically different in both models (Figure S3), protein levels of CAPN2 and CAPNS1 known to have a long half-life, were still high at 28 dppS (Figure 6A–C). However, no difference in total calpain activity could be found between both lactation models at 28 dpp (Figure 6D). As expected, cleavage of the CAPN2-target, E-cadherin was barely detected at 28 dpp either in the long or the short lactation models, indicating the full regression of the mammary gland (Figure 6E).

The model of lactation might induce persistent changes in the mammary tissue. Factors unique to the postpartum mammary gland microenvironment are thought to increase risk in PPBC [23,24].

We examined the extent to which the lactation model induces lasting changes in the mammary gland focusing on the expression of microRNAs. Differential expression of miRs was studied in mammary tissue samples at 28 dpp in both models of lactation. Sample readings were annotated to the miRs included in the Ensembl database and miRs with very low counts across all libraries were filtered out prior to further analysis. A total of 945 miRs were subsequently normalized to eliminate composition biases between libraries following the trimmed mean of M-values (TMM) normalization method [25]. Specific dispersions per gene with a negative binomial distribution were also estimated [26,27]. The analysis of the whole miRNome resulted in six differentially expressed miRs (FDR < 0.05).

**Figure 7:**
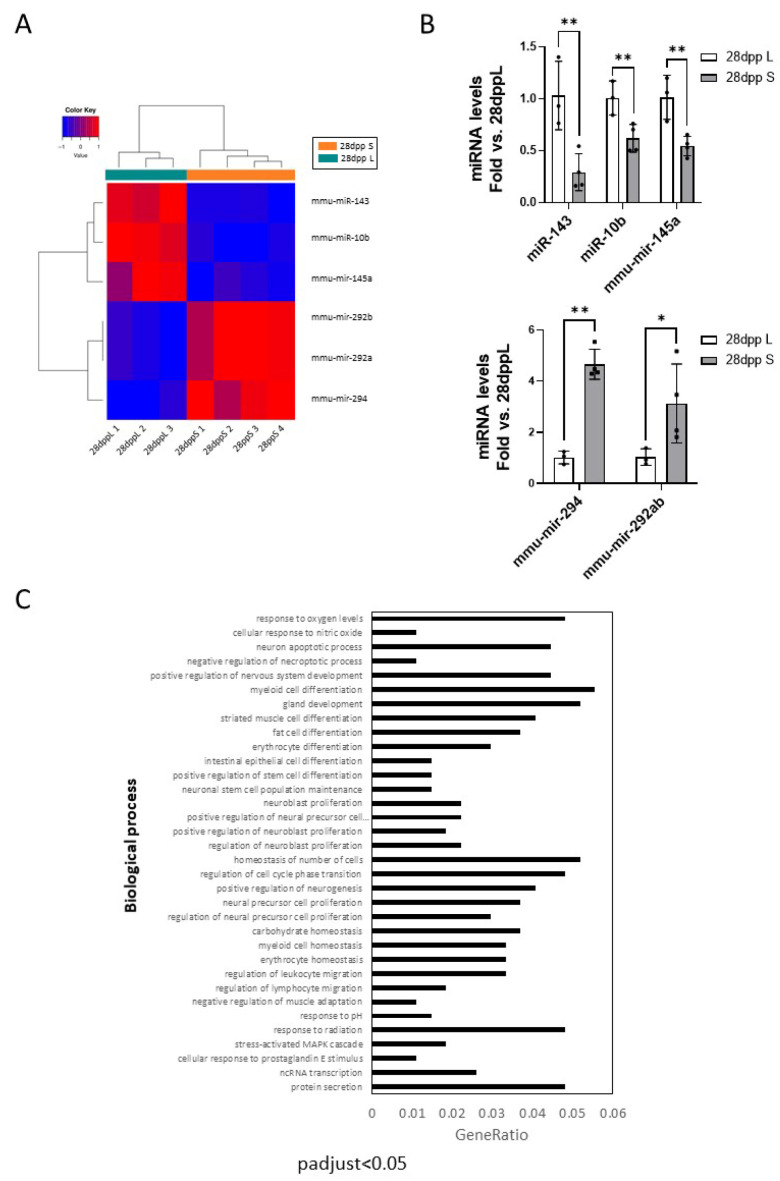
Differential expression of miRNAs in mammary gland at 28 dpp after long/short lactation. (**A**) Heatmap showing the clustering of samples based on logCPM of significantly differentially expressed miRNAs (FDR < 0.05). The logCPM values were transformed and mean-centered to range from −1 to 1. Values close to 1 (red) indicate overexpression in 28 dppS; values close to −1 (blue) indicate under-expression in 28 dppS. (**B**) qPCR validation of downregulated (upper plot) and upregulated (lower plot) miRs in mammary tissue at 28 dppS were represented as fold vs. 28 dppL. * *p* < 0.05 and ** *p* < 0.01. (**C**) Gene ontology (GO) over-representation analysis (ORA) of miRNAs in 28 dppS mammary gland. Functional analyses of significantly downregulated miRs found in 28 dppS samples vs. 28 dppL (*p*.adjust < 0.05). The *y*-axis represents the term where genes are enriched, and the *x*-axis represents the ratio of term genes to the total genes in the pathway.

Samples from both models of lactation (28 dppL and 28 dppS) exhibit a perfect clustering heatmap according to their respective groups, with a clear distinction in the expression patterns between groups (Figure 7A). Differentially expressed miRs included three upregulated (miR-294, *p* = 6.69^−5^; miR-292a/b, *p* = 2.36^−4^) and three downregulated miRs (miR-143, *p* = 6.00^−10^; miR-10b, *p* = 1^−4^ and miR-145a, *p* = 4.27^−6^) at 28 dppS compared to 28 dppL. The differential expression of miRs was validated by RT-qPCR and plotted as fold change versus 28 dppL (Figure 7B). Importantly, the three downregulated miRs have been considered as tumor suppressors, reported to be downregulated in breast cancer [28,29,30,31,32,33,34,35]. Moreover, the anti-inflammatory role of the cluster mir-143/mir-145 has been extensively reported [36]. The GO-ORA enrichment analysis of genes predicted to be targeted by upregulated miRs showed very poor significance; myeloid leukocyte activation was the only pathway significantly enriched in targets of upregulated miRs. In contrast, the target genes of downregulated miRs (Figure 7C) were enriched in biological processes such as cell death, nitric oxide and oxidative stress, cell differentiation and development (including gland development), immune cell migration and cell proliferation, all key processes for the remodeling of mammary gland. These data suggest that crucial pathways for the maintenance of mammary gland architecture and function are not only affected by the model of lactation, but remain altered in fully regressed mammary tissue. Their identification as breast cancer risk factors remains to be determined.

## 3. Discussion

Postpartum breast cancer has recently been recognized as a clinical entity, separate from pregnancy-associated breast cancer. It accounts for approximately 45% of breast cancer cases diagnosed in young women within 10 years after childbirth [24,37,38]. Compared to breast cancer in nulliparous or pregnant women, PPBC is associated with a poorer prognosis [24]. Notably, breastfeeding for 1–2 years has been linked to a 4.3–33% reduction in breast cancer risk, independent of factors such as age, parity, and menopausal status [39]. While the benefits of breastfeeding for children are well documented, the implications of the model of lactation on maternal breast cancer risk need to be further investigated.

A key contributor to PPBC is the tumor-promoting environment of the mammary gland following lactation [1,24]. Most experimental studies use a model of mammary gland involution by forced weaning after short lactation to study the events underlying this process. While this approach ensures experimental consistency, it does not reflect a more physiological model in which pups are spontaneously weaned after long lactation. Although aware that this model does not exactly reproduce the human model, herein we tried to mimic the most frequent breastfeeding models followed by women to study their effect on calpains as regulatory nodes of mammary gland involution. Furthermore, we explored how the model of lactation can impact the differential expression of other regulatory nodes such as miRs after complete regression of mammary gland.

A limitation of this study is the absence of a detailed, day-by-day analysis of the complex regulation of mammary gland involution after spontaneous weaning and a causal relationship between calpains and the affected biological processes. Regardless of this limitation, our findings indicate that the model of lactation significantly influences specific biological processes of involution. Despite similar morphological regression, some molecular pathways differ between models. Either the extension of the response was not the same or the pathways involved in this stage were not equally coordinated in both models.

The long lactation model exhibited a milder second-phase involution, evidenced by altered expression of lactation/involution markers, attenuated inflammatory response, reduced caspase-3 activity, and distinct ECM remodeling. It could be hypothesized that in the long lactation model the transition into the second phase involution was delayed. This hypothesis, however, does not align with data showing the same adipocyte repopulation and epithelium/stroma ratio in both models at 72 hW and 21 dL. Moreover, the limited number of differentially expressed miRs identified in this study indicates a very similar involution process in both models.

Post-lactational involution is a finely regulated multistep process which depends on synchronized regulatory nodes and signaling pathways. Even minor disruptions in timing or node interactions may have exponentially growing consequences for mammary tissue. Interestingly, CAPN1 and CAPN2 dynamics markedly varied between models. In the short lactation model, both isoforms showed higher expression and activity than those observed at any time point of mammary gland involution after spontaneous weaning. Aberrant calpain expression is documented in both breast cancer tissues and cell lines, further supporting their relevance to tumor progression [1,3,13,14,18,40,41]. Disruption or abnormal coordination among regulatory nodes during post-lactation involution has been linked to a metastatic breast cancer signature [41]. We might question what can be considered as “an abnormal coordination” among regulatory nodes. Perhaps the abnormal coordination is found in the mammary gland involution after short lactation by forced weaning compared to the more physiological spontaneous weaning.

We also studied the effect of mammary gland environment on regulatory nodes and their resultant consequences. The inflammatory context appears critical in modulating calpain expression and function in breast tumors [40]. A milder inflammatory response—evidenced by lower IL-6 and TNFα, as well as higher IκBα—in the long lactation model may limit calpain activity. In agreement with this, we already reported the NFκB-mediated induction of CAPN1 and -2 expression during mammary gland involution after forced weaning [9,11]. Consequently, lower NFκB activation might account for lower calpains expression in the long lactation model. Remarkably, in NOS-2 KO mice, calpain-2 appears particularly responsive to inflammation triggered by forced weaning. Interestingly, calpains can also drive inflammation in several tissues and conditions [42]. Thus, future research must investigate bidirectional interactions between calpains and inflammatory pathways.

A different interplay among regulatory nodes likely influences both cell death and matrix remodeling. In forced weaning models, calpain-1 and calpain-2 mediate cell death through the cleavage of isoform-specific targets [11,12,13]. The inhibition of calpain activity in calpeptin-treated mice prevents caspase-3 activity during the second stage of mammary gland involution [11]. In the long lactation model cell death mediated by caspase-3, calpain-2 and calpain-1 is induced in a mild and sustained way, potentially leading to a more effective and less disruptive tissue remodeling process. Accordingly, the epithelium/stroma ratio and the % of adipocyte repopulation are not significantly different between models at 21 dL and 72 hW, further suggesting the efficiency of the cell death process in the long lactation model. It has been hypothesized that immunosuppression and inflammatory cytokines promote survival of epithelial and tumor cells which, not being recognized by the immune system, have a better chance to proliferate and disseminate [24].

Tissue remodeling during involution, resembling wound healing and driven by inflammation, may facilitate tumor dissemination [24]. Increased collagen deposition correlates with an increased risk of breast cancer [42,43]. Furthermore, calpains are known to contribute to ECM remodeling [20,44,45]. We observed higher collagen deposition in 72 hW mammary tissue than in any of the tissue samples from the long lactation model. In agreement with these data, increased collagen deposition has previously been observed in mammary gland from FVB/N mice 56 days after abrupt weaning [4]. Decreased collagen deposition has been reported in murine models of reduced inflammatory environment during involution [19,42], further demonstrating the link between the inflammatory environment and collagen deposition.

In both humans and mice, postpartum involution is characterized by immune cell infiltration, which in women may persist for years, contributing to the increased risk of PPBC [4]. Persistent regulatory node alterations were also observed in the short lactation model even after complete mammary gland regression, suggesting a prolonged tumor-promoting environment. Among these, miR-10b which was downregulated in 28 dppS, has emerged as a potential breast cancer tumor suppressor [29,30,33,34]. Factors such as metabolic disorders can also contribute to the suppression of miR-10b expression in primary tumors [32]; however, its precise role during postpartum involution remains to be fully elucidated.

The miR-143/145 cluster, a known tumor suppressor and anti-inflammatory group, is often downregulated in breast cancer [35]. Furthermore, miR-143/145 have been proposed as potential biomarkers for breast cancer prognosis [31]. Interestingly, miR-145 overexpression has been reported to indirectly decrease calpain-2 levels [46]. Accordingly, the downregulation of miR-145 in 28 dppS mammary samples might be upstream of increased calpain-2 levels.

## 4. Materials and Methods

### 4.1. Materials

Primary and secondary antibodies as well as Taqman primers used in this study are provided in Supplementary Tables S1 and S2.

### 4.2. Animals

All animals were of C57BL/6 genetic background and maintained under controlled conditions (12-h light/12-h dark cycle, 22 ± 2 °C, 50–60% humidity) with ad libitum access to standard chow and water. For specific experiments, NOS-2 constitutive knockout mice on the same genetic background (C57BL/6-Nos2tm1) were used. NOS-2 KO mice were purchased at Taconic bioscience, Inc (Model#: 14897, Seattle, WA, USA).

All experimental procedures were conducted in accordance with the European Directive 2010/63/EU for the protection of animals used for scientific purposes, the National Institutes of Health guidelines, and the Guiding Principles for Research Involving Animals and Humans approved by the Council of the American Physiological Society. Protocols were reviewed and approved by the Ethics Committee for Animal Experimentation of the University of Valencia (protocol code 2022-VSC-PEA-0284). For experiments involving NOS-2 knockout mice, mammary tissue samples were obtained from previously conducted, ethically approved protocols and stored at −80 °C, in compliance with the 3Rs principle to reduce the use of live animals. Virgin female mice (7–8 weeks old) were mated and housed individually throughout pregnancy. After parturition, litter size was standardized to six pups per dam to ensure uniform lactational demand. Mice were randomly assigned to experimental groups (short or long lactation models) using a computer-generated random sequence. Animals were monitored daily by trained personnel for signs of distress or adverse events. Any mice exhibiting illness or abnormal behavior, including cannibalistic behavior, such as consuming their own pups, were excluded from the study.

Two lactation models were established (Figure 1A): short lactation model (forced weaning): At the peak of lactation (day 10 postpartum; 10 dL), pups were permanently removed, and dams were sacrificed either 48 h after weaning (48 hW), at the onset of the second phase of mammary involution (72 hW) or 18 days after weaning at 28 days postpartum (28 dppS). Long lactation model (spontaneous weaning): Female mice remained with their pups until spontaneous weaning, which typically occurred by day 21 postpartum. Dams were sacrificed at 15 dL, 21 dL or after 28 days lactating at 28 days postpartum (28 dppL).

Experiments with NOS-2 KO mice were designed as the short lactation model described above. Wild type and NOS-2 KO female mice were sacrificed at 24 hW, 48 hW and 72 hW.

Female mice were always euthanized by CO_2_ inhalation followed by cervical dislocation, and inguinal mammary glands were collected at the indicated time points (see Figure 1A). Sample size was determined a priori using G*Power 3.1 software, with a significance level of 0.05, statistical power of 0.8, and a minimum detectable effect size of 0.4, resulting in a sample size of 6 animals per group.

Investigators performing histological, molecular, and biochemical analyses were blinded to the lactation group allocation to minimize bias in outcome assessment.

### 4.3. Histological Analysis

Paraffin-embedded tissue sections (3 μm) were stained with hematoxylin and eosin (H&E) for histological analysis as described elsewhere. Collagen deposition was analyzed by Masson’s trichrome staining using the “Artisan Masson’s Trichrome Stain Kit” (AR173, Agilent technologies, Carpinteria, CA, USA) following the manufacturer instructions. Stained tissue sections were scanned with a digital scanner Panoramic 300 flash DX and analyzed with Slide viewer 3D HISTECH.

To quantify the relative areas of epithelium, stroma, and adipocytes, scanned histological images of H&E-stained tissue sections were analyzed using QuPath software (v4.4) as previously described [47]. QuPath is an open source software licensed under the GNU General Public License. In brief, a pixel classifier was trained by manually annotating representative regions of each tissue type (epithelium, stroma, adipose tissue), allowing the software to distinguish them based on color and texture features. The Random Trees classifier, an algorithm implemented in QuPath for pixel classification, was applied to segment the images accordingly. Following classification, the percentage area occupied by each tissue component was calculated within the region of interest. In addition, the stroma-to-epithelium ratio was computed to evaluate relative tissue composition.

For collagen quantification, Masson’s stained tissue sections were also analyzed in QuPath using positive pixel detection. The algorithm was configured to detect collagen-specific staining based on hue, saturation, and intensity thresholds. The percentage of positively stained pixels was calculated within predefined regions of interest.

### 4.4. Gene Expression Analysis by RT-qPCR

Total RNA from mammary tissue was extracted using RNeasy® Mini Kit (Qiagen, Germantown, MD, USA) according to the manufacturer’s protocol. RNA concentration and purity were determined using NanoDrop ND-2000 (NanoDrop Technologies, Wilmington, DE, USA). RNA (500 ng) was reverse-transcribed into cDNA using a high-capacity RNA-to-cDNA kit (Applied Biosystems, Foster City, CA, USA). The cDNA products were amplified by qPCR using the GeneAmp Fast PCR Master Mix (Applied Biosystems) and inventoried Taqman primers (Table S2). All reactions were carried out in triplicate. Results were normalized according to Ipo8 quantification in the same sample reaction. The threshold cycle (Ct) was determined, and the relative gene expression expressed as relative amount = 2 − Δ(ΔCt), where ΔCt = Ct(target) − Ct(18 S), and Δ(ΔCt) = ΔCt (weaned) − ΔCt (control).

### 4.5. Total Protein Extraction and Immunoblotting

Total protein was extracted in RIPA buffer supplemented with protease and phosphatase inhibitors. Equal amounts of protein were size-fractionated by SDS-PAGE gel electrophoresis and electroblotted onto nitrocellulose membranes (Protran, Whatman). Membranes were blocked and incubated with the specific primary and HRP-conjugated secondary antibody (Table S1). Blots were developed by enhanced chemiluminescence reaction (Cytiva. Amersham™ ECL™ Western Blotting Detection Reagent. Waukesha, WI, USA). Band-intensity was quantified with ImageJ 1.54p. Equal loading was confirmed by re-probing blots with GAPDH or β-actin antibodies. To avoid membrane stripping whenever possible, blots were cut and each strip incubated with different antibodies to detect several proteins in the same membrane. All time-course samples were run with the same control samples on the same gels. To quantify protein levels, we always compared experimental samples to the same control samples.

### 4.6. CAPN Activity Assay

CAPN activity was measured as described [11] using the ‘calpain activity assay kit’ (QIA-120, Merck KGaA, Darmstadt, Germany) according to the manufacturer’s instructions. Briefly, total protein extracts from mammary gland samples were prepared using cell lysis buffer (CytoBuster™ Protein Extraction Reagent, Merck KGaA). Mammary gland samples (30 μg) and standards were incubated in the presence or absence of BAPTA inhibition buffer, with activation buffer (supplemented with Ca^2+^ and the reducing agent TCEP) and the substrate (Suc-Leu-Leu-Val-Tyr-AMC) supplied in the kit. After 15 min, fluorescence was measured on a plate reader (Molecular Devices SPECTRAmax Gemini XPS, San Jose, CA, USA)at an excitation λ360–380 nm and emission λ440–460 nm. CAPN activity was calculated as the difference between the signal obtained in the presence of inhibition buffer (BAPTA) and that measured with activation buffer alone.

### 4.7. Caspase-3 Activity Assay

Caspase-3 activity was measured in mammary tissue lysates using the ‘Caspase-3/CPP32 Colorimetric Assay Kit’ (#K106, BioVision, R&D Systems, Mountain View, CA, USA) as previously described [11]. Briefly, 50 μg of total protein extracts from mammary gland solubilized in cell lysis buffer were incubated with DTT reaction buffer and the labeled p-nitroaniline substrate (DEVP-pNA) for 1 h at 37 °C. The chromophore p-nitroaniline (pNA) after cleavage from the labeled substrate DEVD-pNA was detected at plate reader (Molecular Devices SPECTRAmax Gemini XPS, San Jose, CA, USA) at a emission λ = 405 nm.

### 4.8. miRnome Analysis

The detailed methods for miRnome analysis are provided in Supplementary Material. Small RNA sequencing was performed at the CIBER-ISCIII INCLIVA Biomedical Research Center core facility. Processing and comprehensive small RNA-seq data analysis was carried out by Epidisease S. L. (spin-off from the INCLIVA). Briefly, cell-free total RNA (including miRs) was isolated from tissue samples and quantified. After library amplification, the library product was purified and single-end sequencing performed on Illumina NextSeq550 platform (Illumina, San Diego, CA, USA) as described. Raw FASTQ files were processed following standard methodologies. The normalization of read counts per miRNA per sample was performed using the TMM (Trimmed Mean of M-values) method, as implemented in the edgeR package v4.6.3. The average quality of the reads was assessed.

The differential expression of miRs between 28 dppS and 28 dppL was analyzed in mammary tissue samples. Functional analysis of target genes for statistically different miRs was performed by ORA (over-representation analysis, FDR < 0.05).

### 4.9. qPCR Validation of Differentially Expressed miRNAs

Reverse transcription reactions were performed using the TaqMan MicroRNA Reverse Transcription kit (Part No. 4366596, Applied Biosystems.Inc., Foster City, CA, USA), miR-specific stem-loop primers and 100 ng of cell-free RNA in a 20 μL RT reaction. Conditions for retrotranscription were 16 °C for 30 min, 42 °C for 30 min and, finally 85 °C for 5 min.

Real-time qPCR reactions were performed in triplicate using 5 μL TaqMan 2× Universal PCR Master Mix with No UNG (Applied Biosystems), 0.5 μL TaqMan Small RNA assay (20x) (Applied Biosystems), 3.5 μL of nuclease free water and 1 μL of RT product. Real-time PCR was carried out on a QuantStudio 5 Real-time PCR System (ThermoFisher Scientific, Waltham, MA, USA) programmed as follows: 50 °C for 2 min and 95 °C for 10 min followed by 45 cycles of 95 °C for 15 s and 60 °C for 1 min.

Selected miRs were validated using TaqMan™ MicroRNA Assay (Applied Biosystems) referenced in Table S2. The snoRNA-234 was used as an endogenous control for the normalization analyses and fold-change data were obtained using the delta-delta Ct method (2^−ΔΔCt^).

### 4.10. Statistical Analysis

The statistical analysis and graph plotting were undertaken using the Graph Pad Prism software v.10.4.1. Data were analyzed by a one-way Anova test with multiple comparisons and then by Tukey’s test. The results were expressed as mean  ±  standard deviation, and differences were considered significant when *p*  ≤ 0.05 (*), *p*  ≤ 0.01 (**). Independent experiments were conducted with a minimum of three replicates per condition to allow statistical comparison.

## 5. Conclusions

Our results underscore the importance of regulatory node dynamics for mammary gland biology, and highlight how a milder inflammatory response after long lactation might prevent persistent molecular changes relevant to PPBC. Future research on PPBC risk should explore abnormal patterns within these nodes to unveil molecular signatures for risk assessment and therapeutic strategies in PPBC. Importantly, these studies should prioritize physiologically relevant models to better replicate human lactation patterns. Clarifying how lactation influences breast cancer risk will be essential for promoting informed, evidence-based breastfeeding practices beneficial for women’s long-term health.

## Data Availability

The original contributions presented in this study are included in the article/Supplementary Material. Further inquiries can be directed to the corresponding author.

## Supplementary Materials

The following supporting information can be downloaded at https://www.mdpi.com/article/10.3390/ijms262110501/s1.

## Abbreviations

The following abbreviations are used in this manuscript:

CAPN	Calpain
dL	Days of lactation
dpp	Days post-partum
hW	Hours after forced weaning
IκBα	Inhibitor of kappa B
L/S	Long or Short model of lactation
MMP	Metalloproteinase
NFκB	Nuclear Factor kappa B
NOS-2	Nitric oxide synthase-2
PPBC	Post-partum breast cancer
TIMP-1	Tissue inhibitor of metalloproteinase-1
TNF-α	Tumor necrosis factor-α
