# Supplementary material for: The Length of Lactation and Model of Weaning Modulate Key Regulatory Nodes of Murine Mammary Gland Involution
Source: Int J Mol Sci. 2025 Oct 29;26(21):10501. doi: 10.3390/ijms262110501 (PMC12607435; doi:10.3390/ijms262110501)
Supplement: Supplementary file 1 [file ijms-26-10501-s001.zip › ijms-3867934-supplementary.pdf]

The length of lactation and model of weaning modulate key regulatory nodes of murine mammary gland involution.

Sara Puebla[1,2], Amparo Gimeno[3], Elena Ortiz-Zapater[1,2], Rosa Zaragozá[1,3], Juan R Viña[1,2], Elena R. García-Trevijano[1,2#]

1 Departamento de Bioquímica y Biología Molecular. Facultad de Medicina. Universidad de Valencia. Spain

2 Fundación Investigación Hospital Clínico-INCLIVA. Valencia. Spain

3 Departamento de Anatomía y Embriología Humana. Facultad de Medicina. Universidad de Valencia. Spain

# Corresponding author: E.R. García-Trevijano

Departamento de Bioquímica y Biología Molecular. Fundación Investigación Hospital Clínico-INCLIVA. Facultad de Medicina. Universidad de Valencia. Spain

Avda. Blasco Ibañez, 15. 46010 VALENCIA. SPAIN

Pho.: 0034 963 683 886

Fax: 0034 963 864 001

E-mail address: elena.ruiz@uv.es

ORCID: 0000-0002-3985-1270

SUPPLEMENTARY METHODS

miRnome analysis

RNA extraction and quantification. Cell-free total RNA (including miRs) was isolated from tissue samples using the miRvana miRNA isolation kit (Invitrogen, Thermofisher, Waltham, MA, USA), following the manufacturer's protocol. Concentration of total cell-free RNA (including miRs) was quantified using NanoDrop™ One Microvolume UV–Vis Spectrophotometer (Thermo Scientific Inc, Waltham, MA, USA).

Library preparation and small RNA-sequencing. Small RNA libraries were generated and indexed using the Lexogen's Small RNA-Seq Library Prep Kit for Illumina sequencing (Lexogen GmbH Campus Vienna Biocenter 5, 1030 Vienna, Austria). Briefly, for library generation 3 '-adapter was ligated to RNA and their excess removed by column purification. Next, 5'-adapter was ligated and the RNA, flanked by 5 'and 3' adapters, was converted into cDNA. After library amplification and External i7 indexes, the library product was purified and single-end sequencing performed on Illumina NextSeq550 platform (Illumina, San Diego, CA, USA) on High Output 1 × 50pb RUN (NextSeq 500/550 High Output v2 75 cycles kit, FC-404-2005).

Preprocessing, Quality Control, and Normalization. Pipelines used in this study were: FastQC, BBduk, subread, bowtie2, multiqc, edgeR, limma, and limma-voom. miRNAs present in the samples were mapped to the mouse reference genome (GRCm39) using two independent aligners (subread and bowtie2) and annotated based on the Ensembl database. All miRNAs with zero counts across all samples in this cohort were removed. Raw FASTQ files obtained from sequencing were processed to remove sequencing adapters and to trim the reads based on length, following standard methodologies commonly applied to this type of data. Normalization of read counts per miRNA per

sample was performed using the TMM (Trimmed Mean of M-values) method, as implemented in the edgeR package. The average quality of the reads was assessed by the mean Phred Quality Score obtained both before and after preprocessing. On average, each sample contained 6.50 million sequences prior to preprocessing and 6.12 million afterward, indicating that a low number of discarded sequences. A multi-dimensional scaling (MDS) plot was used to analyze sample distribution according to miRs expression values.

Prediction of miRNA targets and over-representation analysis. Four packages (edgeR, voom, limma-voom and deseq2) were used to analyze differential expression of miRs between 28dppS and 28dppL mammary tissue samples. Functional analysis of target genes for statistically different miRs was performed by ORA (over-representation analysis, FDR < 0.05).

Supplementary table S1. List of antibodies used in this study

PRIMARY ANTIBODY	REFERENCE
**CAPN1**	Abcam, ab3170
**CAPN2**	Cell Signaling, CS2539
**CAPNS1**	Abcam ab28237
**Stat5a**	Abcam ab32043
**pStat3**	Cell Signaling, CS9145
**Stat3**	Cell Signaling, CS4904
**E-cadherin**	BD Biosciences, 80182
**Mab414**	Biolegend, MMS-120P
**IκBα,**	Cell Signaling , CS4814
**β-actin**	Abcam, ab8227
**GAPDH**	Abcam ab8245

SECONDARY ANTIBODY	REFERENCE
Anti-Rabbit Immunoglobulins/HRP	Dako, P0448
Anti-Mouse Immunoglobulins/HRP	Dako, P0447

Supplementary table S2. List of primers used in this study

GENE	REFERENCE
**CAPN1**	Mm00482964_m1
**CAPN2**	Mm01321635_m1
**CAPNS1**	Mm00501568_m1
**IL-6**	Mm00446190_m1
**TNF[illegible]**	Mm00443258_m1
**MMP2**	Mm00439506_m1
**MMP9**	Mm00442991_m1
**TIMP-1**	Mm01341361_m1
**Ipo8**	Mm01255158_m1
**mmu-miR-145***	Assay ID: 002514
**mmu-miR-143**	Assay ID 002249
**mmu-miR-294**	Assay ID: 001056
**mmu-miR-292-5p**	Assay ID: 001055
**mmu-miR-10b***	Assay ID: 002572
**snoRNA-234**	Assay ID: 001234

SUPPLEMENTARY FIGURES

Supplementary Figure 1

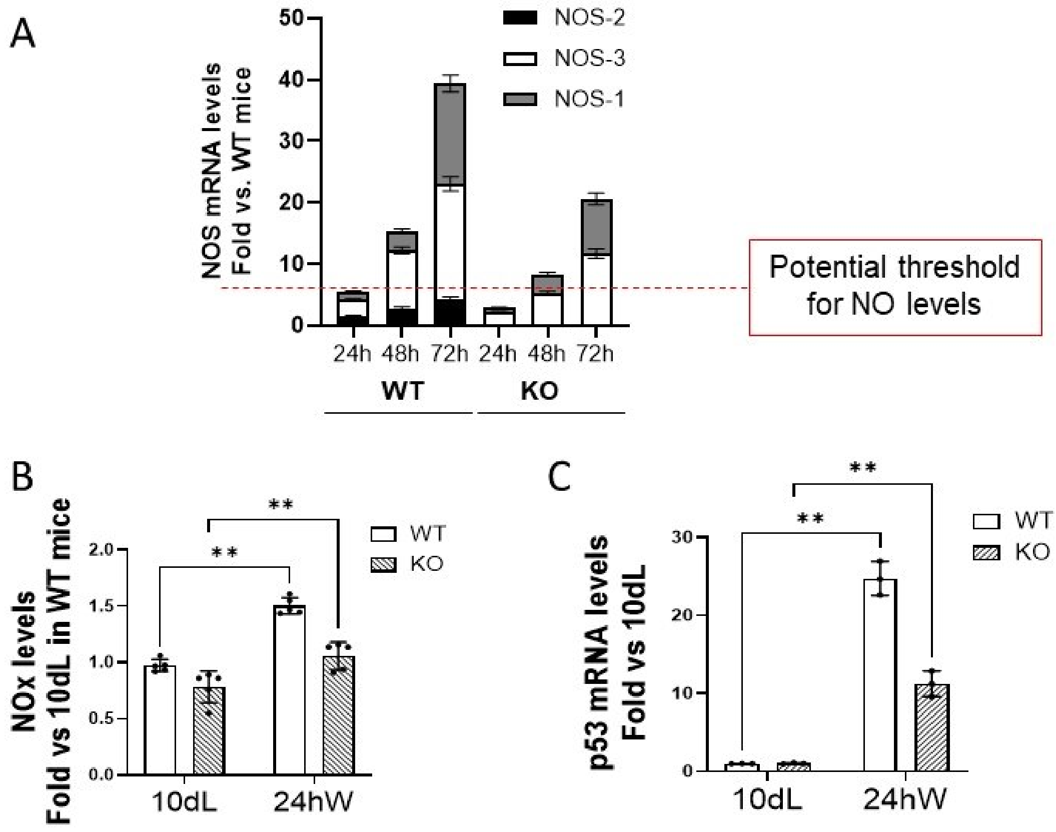

Figure S1. Depletion of NOS-2 leads to 24h delayed mammary gland involution. A) NOS-1, NOS-2 and NOS-3 mRNA levels in mammary gland after 24, 48 and 72h of forced weaning analyzed by qPCR in WT and NOS-2 KO mice. The expression of the three NOS isoforms is plotted as fold vs 10dL WT mice $p<0.01$. B) NOx levels and p53 mRNA levels C) in 10dL and 24hW mammary gland samples from WT and NOS-2 KO mice. Normalized data (n=3) were plotted as fold vs 10dL. ** $p<0.01$.

The expression of the three NOS isoforms is induced in a time-dependent manner after forced-weaning in WT mice. NOS-1 and NOS-3 expression is induced in KO as in WT mice. The NO content in mammary gland is expected to result from the additive contribution of the three NOS isoforms. The NO threshold which most likely triggers a response in mammary gland is indicated as a red line. The NO threshold to trigger a full response for the first stage of mammary gland involution is reached at 24hW in WT mice; however, in KO mice this NO content is not reached until 48hW. The first stage of mammary gland involution is a p53-dependent and reversible phase. Accordingly, p53 mRNA levels increase in both, WT and NOS-2 KO mice at 24hW; however, this increase is dramatically reduced in KO mice, explaining the 24h delay in the downstream response reported for these animals.

Supplementary Figure 2

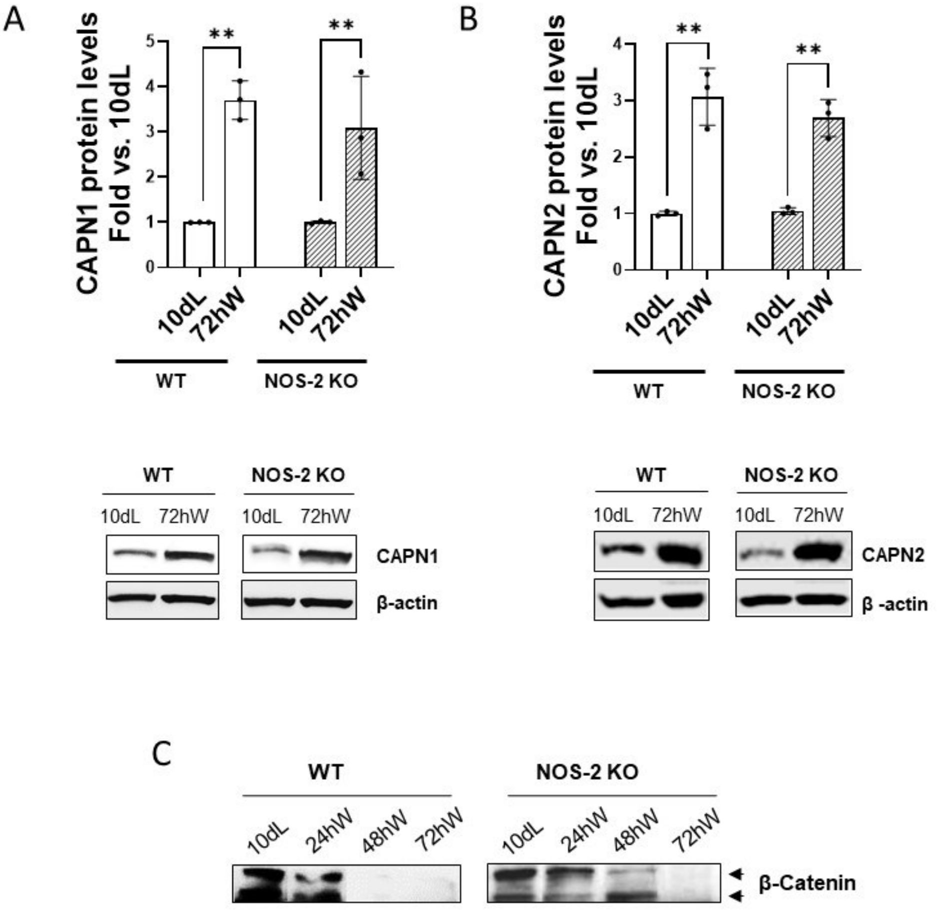

Figure S2. Role of inflammation on calpains expression analyzed in WT and NOS-2 KO mice. CAPN1 A) and CAPN2 B) protein levels in mammary gland at 10dL and 72hW analyzed by western blot in WT and NOS-2 KO mice. Normalized data (n=3) were plotted as fold vs 10dL. ** $p<0.01$. Protein levels of both CAPNs in mammary gland from WT and KO mice are induced at 72hW. C) Cleavage of the CAPN2-target, β-catenin analyzed by western blot in 10dL, 24h, 48h and 72hW mammary gland samples from WT and NOS-2 KO mice.

While still detected at 48hW in mammary samples from KO mice, a full proteolysis of β-catenin was observed in WT mice. The delayed expression/activity of CAPN2 in KO mammary gland has functional consequences during mammary gland involution.

Supplementary figure 3

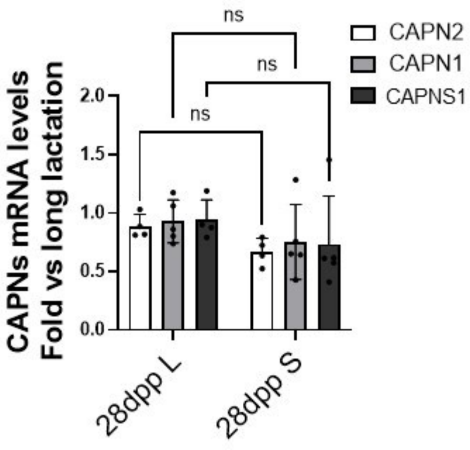

Figure S3. mRNA levels of CAPN1, CAPN2 and CAPNS1 at 28 days postpartum in different models of lactation. mRNA levels of CAPN1, 2 and S1 were analyzed by qPCR in mammary tissue samples 28dppL and 28dppS. Data were quantified, normalized by the expression of Ipo8 and plotted as Fold vs 28dppL No statistical differences were found.
